# Molecules and mechanisms that regulate multipolar migration in the intermediate zone

**DOI:** 10.3389/fncel.2014.00386

**Published:** 2014-11-14

**Authors:** Jonathan A. Cooper

**Affiliations:** Fred Hutchinson Cancer Research Center, Division of Basic SciencesSeattle, Washington, USA

**Keywords:** neuron migration, axonogenesis, multipolar migration, neocortex development, radial migration, cortical lamination, neuron locomotion, mini-columns

## Abstract

Most neurons migrate with an elongated, “bipolar” morphology, extending a long leading process that explores the environment. However, when immature projection neurons enter the intermediate zone (IZ) of the neocortex they become “multipolar”. Multipolar cells extend and retract cytoplasmic processes in different directions and move erratically—sideways, up and down. Multipolar cells extend axons while they are in the lower half of the IZ. Remarkably, the cells then resume radial migration: they reorient their centrosome and Golgi apparatus towards the pia, transform back to bipolar morphology, and commence locomotion along radial glia (RG) fibers. This reorientation implies the existence of directional signals in the IZ that are ignored during the multipolar stage but sensed after axonogenesis. *In vivo* genetic manipulation has implicated a variety of candidate directional signals, cell surface receptors, and signaling pathways, that may be involved in polarizing multipolar cells and stabilizing a pia-directed leading process for radial migration. Other signals are implicated in starting multipolar migration and triggering axon outgrowth. Here we review the molecules and mechanisms that regulate multipolar migration, and also discuss how multipolar migration affects the orderly arrangement of neurons in layers and columns in the developing neocortex.

## Introduction and scope

The neocortex develops by the coordinated migration of projection neurons from the neocortical ventricular zone (VZ) and interneurons from the ganglionic eminences (Hatten, 2002; Marín and Rubenstein, 2003). While projection neurons move generally outwards from the VZ to the top of the cortical plate (CP), live imaging of individual cells has revealed that their radial progress is interrupted by an extended period of random migration. During this time the neurons appear to be “stellate” or “multipolar” (MP), characterized by multiple (>3) cytoplasmic projections that point in different directions. The primary cilium and centrosome are oriented randomly relative to the pial surface. MP cells migrate with frequent changes of direction, moving sideways (tangentially), up (towards the pia) or down (towards the VZ). Axons are initiated during the MP phase. MP neurons are quite different from migrating neurons in other brain regions, which generally are elongated in the direction of travel and have a prominent leading process. MP migration can lead to horizontal dispersion of neurons, and may be functionally significant for forming cortical circuits. In addition, the duration of MP migration differs from neuron to neuron, which has implications for cortical layering. Finally, the signaling mechanisms that initially cause polarized cells from the VZ to become MP, and which cause MP cells to resume radial migration and become bipolar, remain mysterious despite intense study.

This review addresses new developments and continuing uncertainty regarding the external signals and intracellular mechanisms that regulate MP cells at three important times: when MP migration starts, when the axon starts to grow, and when MP cells repolarize towards the pia and resume radial migration with bipolar morphology. The review also discusses the implications of MP migration for the radial unit hypothesis of cortical wiring and for cortical lamination. The focus is on newer research from the last 5–10 years, with an emphasis on loss of function studies. Readers are referred to excellent reviews for earlier work (Bielas et al., 2004; LoTurco and Bai, 2006; Ayala et al., 2007).

## Background: phases of migration of neocortical projection neurons

**Figure 1:**
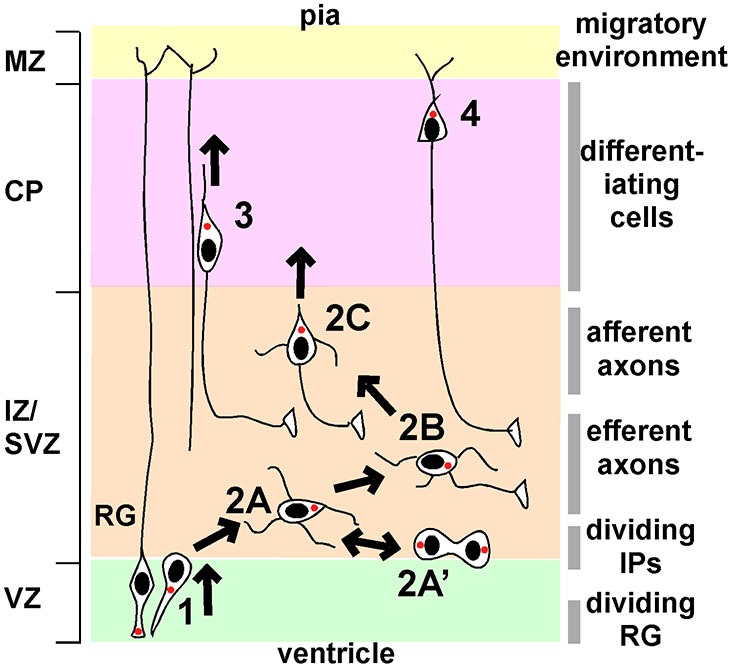
**Phases of migration of neocortical projection neurons.** Principal phases of migration: **(1)**, movement from the VZ to the IZ/SVZ with bipolar or “pin-like” morphology; **(2)**, multipolar migration; **(3)**, bipolar locomotion along radial glia (RG); **(4)**, terminal translocation. Multipolar migration can be subdivided into further stages: **2A**, initial multipolar migration of newborn post-mitotic neurons and intermediate progenitors; **2A’**, division of intermediate progenitors followed by multipolar migration; **2B**, axon emergence and growth; **2C**, stabilization of a dominant pia-directed leading process. The left-hand side shows the principal divisions of the cortex (VZ, ventricular zone; IZ/SVZ, intermediate/subventricular zone; CP, cortical plate; MZ, marginal zone). The right-hand side indicates the major features of the local environment through which neurons migrate. See text for details.

Four phases of projection neuron migration have been described through detailed histological and live imaging studies (Shoukimas and Hinds, 1978; O’Rourke et al., 1992; Nadarajah et al., 2001; Hatanaka and Murakami, 2002; Tabata and Nakajima, 2003; Hatanaka et al., 2004, 2009; Noctor et al., 2004; Ochiai et al., 2007; de Anda et al., 2010; Namba et al., 2014; Figure 1). In phase 1, asymmetric division of radial glia progenitors (RG) in the VZ creates new post-mitotic neurons and intermediate progenitors (IP). These cells exit the VZ with bipolar or “pin-like” morphology. Phase 2 starts when cells reach the subventricular zone (SVZ)/IZ and become multipolar (Figure 1, stage 2A). MP IP divide in the SVZ and their daughters resume MP migration (stage 2A’). After a day or more in the MP phase, a ventricle- or horizontally-oriented process near the centrosome begins to extend and becomes the axon (stage 2B). Phase 2 ends when the MP cell reorients the Golgi and centrosome towards the pia, establishes a dominant pia-directed leading process, and starts radial migration, trailing the axon behind (Hatanaka et al., 2004; de Anda et al., 2010) (stage 2C). This is known at the multipolar to bipolar (MP-BP) transition, and requires the stabilization of a dominant leading process and the correct orientation of that process towards the pia. After the MP-BP transition, neurons rapidly exit the IZ by locomotion along RG (phase 3), followed by phase 4, terminal translocation to the top of the CP.

The principal signaling pathways implicated in these major transitions are summarized in Figure 1 and described in more detail below. Many additional molecules are required for the multipolar to bipolar transition but their regulation by external signals is unclear. These molecules are very briefly reviewed in Box 1.

Box 1 **GTPases, protein kinases and cytoskeletal proteins implicated in the transition from multipolar to bipolar migration.**

In addition to the principal signaling pathways discussed in the main text, the following genes and proteins are implicated in regulating the transition from MP migration to BP migration in the CP. It is not clear whether they are regulated by external signals: they may be cell-intrinsic, or controlled by a transcription program set in motion at the time of neurogenesis.

The protein kinase **Mst3/Stk25** regulates MP migration, apparently by inhibiting RhoA (Tang et al., 2014). Mst3-deficient cells accumulate in the IZ with rounded morphology. Mst3 contains a site for phosphorylation by Cdk5. Cdk5 phosphorylates Mst3 *in vitro*, and Mst3 phosphorylation is reduced in Cdk5−/− brain. Kinase-defective and non-phosphorylated mutants of Mst3 fail to rescue migration. This suggests that Cdk5 phosphorylation and activation of Mst3 is required for migration out of the IZ. *In vitro*, Mst3 over-expression reduces RhoA GTP loading. Remarkably, Mst3 directly phosphorylates RhoA and a non-phosphorylated RhoA mutant has increased GTP loading. This suggests that Mst3 directly inhibits RhoA. Over-expressed RhoA causes IZ arrest, and RhoA knockdown rescues Mst3-inhibited neurons, consistent with Mst3 activation by Cdk5 inhibiting RhoA and permitting exit from the IZ. However, it is not known whether Mst3 activity and RhoA phosphorylation change during migration.

**Rnd2** is an unusual GTPase that is regulated primarily by expression level. Rnd2 expression is induced soon after neurons leave the VZ by proneural gene Neurog2 and maintained in MP cells by NeuroD1 (Heng et al., 2008). Neurog2 and Rnd2 are required for the MP-BP transition, and the requirement for Neurog2 can be by-passed if Rnd2 is artificially expressed (Hand et al., 2005; Heng et al., 2008). The main function of Rnd2 appears to be as an inhibitor of **RhoA**. Accordingly, neurons lacking Neurog2 can be rescued by inhibiting RhoA. The results imply that Rnd2 inhibits RhoA during MP migration and high RhoA activity delays the MP-BP transition.

A requirement for inhibition of RhoA by Rnd2 seems to be in conflict with the requirements for activation by Mst3 and by PlxB2 (see main text) (Azzarelli et al., 2014). How can we reconcile these findings? It is possible that cycling of RhoA between GTP and GDP states is required, or activity be increased in some parts of the cell and inhibited in others. Alternatively, it is possible that some intermediate level of RhoA activity is permissive for radial migration.

**GSK3** is important for neuron polarization and axon growth and branching *in vitro* (Hur and Zhou, 2010). In bipolar neurons, activated GSK3β localizes the microtubule plus-end binding protein adenomatous polyposis coli (APC) to the distal ends of microtubules in the tip, allowing centrosomal forward movement and neuronal migration (Asada and Sanada, 2010). Morgan-Smith et al examined cortical lamination by use of mice lacking GSK3α and deleting GSK3β in early postmitotic neurons with NeuroD6-Cre (Morgan-Smith et al., 2014). While lower neuron layers were normal, upper layers were dispersed, suggesting defects in MP migration or locomotion along RG. When Cre was electroporated into the VZ at E15, GSK3-deficient neurons became arrested in the IZ with MP morphology. Axons were present, but branched abnormally. Phosphorylations of DCX at Ser327 and CRMP2 at Thr514 were inhibited. GSK3 also regulates the canonical Wnt signaling pathway, but mutation of β-Catenin or triple mutation of all Disheveled family members with NeuroD6-Cre did not lead to gross layering defects.

Rab GTPases regulate membrane traffic. When endocytosis is inhibited with dominant-interfering **Rab5** or Rab5 knockdown, some neurons accumulate in the IZ with abnormal, rounded morphology (Kawauchi et al., 2010). However, other neurons had bipolar morphology but were stalled at the bottom of the CP with an abnormally thick trailing process. Co-culture experiments suggested that Rab5-inhibited neurons bind more tightly to RG. Rab5-inhibited cells have a small increase in surface NCad, and NCad knockdown partially rescues their migration. This suggests that abnormally high NCad after the MP-BP transition may inhibit RG-dependent locomotion.

In contrast with Rab5’s role in endocytosis, **Rab11** regulates membrane recycling to the cell surface. Dominant-interfering Rab11 reduces NCad on the cell surface and increases NCad in recycling endosomes (Kawauchi et al., 2010). Neuron migration is also slowed, although the exact stage was not determined. If the delay is in the IZ, then the results would be consistent with Rab11 mediating the Reelin-dependent NCad exocytosis in the MP stage, and Rab5 mediating NCad endocytosis to allow locomotion.

**Cdk5** is a protein kinase that is related to cell cycle kinases but is expressed and functions in non-mitotic cells, including neurons. Cdk5 is required for the MP-BP transition (Ohshima et al., 2007). Cdk5-deficient cells extend axons but remain multipolar and retarded in the IZ. When cells do enter the CP, their leading processes are often branched, suggesting problems with stabilizing a single leading process (Hatanaka et al., 2004; Ohshima et al., 2007). A similar phenotype was noted for cells lacking the Cdk5 activator, p35 (Gupta et al., 2003). Cdk5 phosphorylates many proteins involved in MP migration, including DCX, Ndel1 and p27kip1 (reviewed by Ayala et al., 2007) and axin (Fang et al., 2011). A key question for Cdk5 is whether it is dynamically regulated by external signals during MP migration or is constitutively active. The serotonin 6 receptor, **5HT6R**, was recently reported to regulate MP exit (Jacobshagen et al., 2014). The authors suggested that 5HT6R may work through Cdk5, because 5HT6R binds to Cdk5 and the migration of 5HT6R-deficient cells was partly rescued by over-expressing Cdk5 and its activator, p35 (Jacobshagen et al., 2014). Unfortunately, there is no evidence that serotonin or other extracellular 5HT6R ligands regulate migration.

The role of the **Jnk1** pathway is controversial. Jnk1 activity, measured using antibodies to phospho-Jnk, is high in the IZ, where the Jnk1 activating kinase,** DLK** is highly expressed (Hirai et al., 2002; Kawauchi et al., 2003). Deletion of the DLK gene decreases Jnk1 activity, inhibits axonogenesis, and slows neurons at the IZ-MZ boundary (Hirai et al., 2006). DLK is a member of the Mixed Lineage Kinase family, and like other family members can be activated by Rac1. This suggests that Jnk may be activated by Rac1 via DLK. Indeed, dominant-interfering **Rac1** inhibits Jnk activation *in vivo* (Kawauchi et al., 2003). In turn, Rac1 may be activated by Rac1 GEFs **STEF** and **Tiam1** which are highly expressed in the IZ and CP (Kawauchi et al., 2003). Accordingly dominant-interfering STEF/Tiam1, dominant-interfering Jnk1, or Jnk inhibitors all stall neurons in the IZ, suggesting that a STEF/Tiam1-Rac1-DLK-Jnk1 pathway is involved in IZ exit. The phenotypes of the inhibited neurons are not consistent with a simple, linear pathway, however. Rac1-inhibited neurons are rounded, with reduced processes, suggesting a general defect in process extension and a failure of radial polarization. In contrast, Jnk1-inhibited neurons have undergone the MP-BP transition but the leading process is twisted and irregular (Kawauchi et al., 2003). This suggests that Rac1 regulates MP migration through several effectors, and that Jnk1 is involved in locomotion of BP neurons out of the IZ.

A different conclusion was drawn by Westerlund et al., who found that **Jnk1** is an inhibitor, not an activator, of the MP-BP transition (Westerlund et al., 2011). They found that Jnk1 knockdown or gene deletion stimulates axon outgrowth, IZ exit and migration through the CP. The mechanism appears to involve the neuron-specific Stathmin family member **SCG10**. SCG10 stabilizes microtubules when it is phosphorylated by Jnk1. *In vivo*, SCG10 knockdown or expression of non-phosphorylated mutant SCG10 stimulates axon outgrowth and IZ exit (Westerlund et al., 2011). Tyrosinated (unstable) tubulin is increased in Jnk1−/− cortex. Expressing phospho-mimetic mutant SCG10 in Jnk1 knockdown neurons restores the normal, slow, exit from the MP zone. This leads to a model in which Jnk1 phosphorylates SCG10, stabilizes microtubules and inhibits the microtubule remodeling required for axon outgrowth and for the MP to BP transition. It is not clear how to reconcile this study with those of Kawauchi et al. (2003) and Hirai et al. (2006).

**srGAP2**: srGAP2 contains an F-BAR membrane-bending domain and a GAP domain specific for Rac1. Knockdown of srGAP2 decreases the number of neurons in the IZ and lower CP, suggesting that endogenous srGAP inhibits IZ exit and BP locomotion through the CP (Guerrier et al., 2009). Rapidly migrating srGAP-deficient neurons in the CP have a less-branched leading process than normal. Endogenous srGAP2 may thus inhibit formation of a single leading process. Accordingly, over-expressed srGAP2, or just the F-BAR domain, inhibits the MP-BP transition. However, it is not known if srGAP2 activity changes during migration.

**Kinesin6**: Kinesin6 is a plus-end directed microtubule motor that also binds actin. Kinesin6 knockdown inhibits the MP-BP transition (Falnikar et al., 2013). Kinesin6 crosslinks and slides antiparallel microtubules in the mitotic spindle. Similarly, in differentiating neurons, kinesin6 helps establish and maintain antiparallel microtubules in the dendrites (Lin et al., 2012). In polarized migrating neurons, kinesin6 concentrates near the centrosome in the base of the leading process, potentially helping maintain mixed orientation microtubules in the leading process and concentrating actin in this region (Falnikar et al., 2013).

**FGF13** is an FGF-homologous family (FHF) member that is not secreted, but that acts inside the cell through mechanisms that are receptor-independent. Wu et al showed that FGF13 localizes in growth cones of cultured neurons, binds to microtubules, and stimulates microtubule polymerization (Wu et al., 2012). FGF13-deleted neurons have defective axon outgrowth *in vitro*. *In vivo*, FGF13 knockout delays MP neurons in the IZ, and those neurons that do enter the CP have an excessively branched leading process. Knockdown of FGF13 *in utero* causes a similar MP delay, which is rescued by wildtype FGF13 but not by a multi-alanine mutant that does not bind microtubules. In many ways, FGF13 resembles DCX, which is also a + end microtubule stabilizer that is enriched at the leading edge. Indeed, DCX knockdown causes IZ delay, which is partly rescued by FGF13 over-expression. Reciprocally, FGF13 knockdown is partly rescued by DCX over-expression, suggesting that DCX and FGF13 function in parallel.

## The start of MP migration

**Figure 2:**
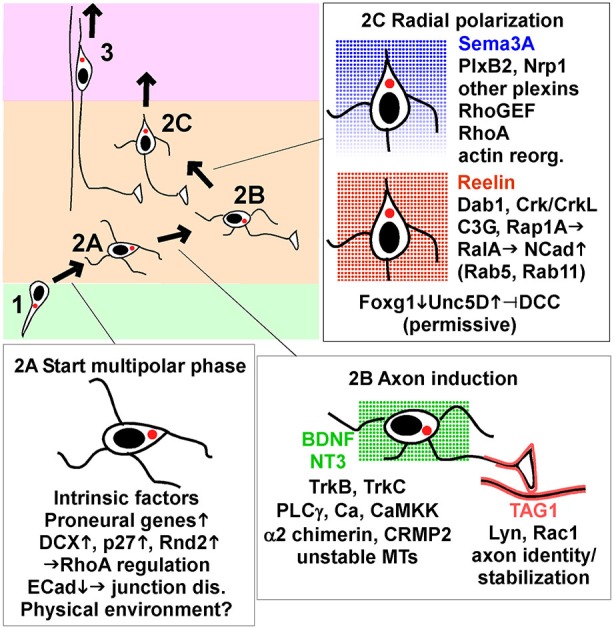
**Key signaling events that start, guide and end multipolar migration and axon induction.** Intrinsic factors and signaling pathways implicated in the movement from VZ to start MP migration (stage 2A), induce and stabilize axons (stage 2B), and induce radial polarization and commence radial migration towards the pia (stage 2C). The insets show a subset of the genes and pathways discussed in detail in the text, focusing on processes that appear to be dynamically regulated by intrinsic transcription changes or signals from the environment. Reelin and neurotrophins BDNF and NT3 are shown as non-directional signals, while Sema3A is thought to form a gradient and provide a direction signal. TAG1 is a protein displayed on the surface of axons in the lower IZ. Abbreviations: junction dis., junction disassembly; actin reorg., actin reorganization; up arrow, increase; down arrow, decrease.

Neurons become multipolar at the boundary between the VZ and SVZ/IZ (Tabata and Nakajima, 2003; Noctor et al., 2004; Figure 2, stage 2A). It is not clear whether this transformation is active or passive. The BP-MP transition could be actively induced by signals present in the SVZ/IZ, but such signals have not been identified. Alternatively, physical interactions could induce the morphological change. The IZ contains a dense neuropil of horizontally-packed axons that may be a barrier to radial migration of BP cells. This region is also the stiffest part of the developing brain (Iwashita et al., 2014). Matrix stiffness influences the morphology and migration behavior of mesenchymal cells, and may similarly affect neurons (Roca-Cusachs et al., 2013). Alternatively, cells may transform to MP morphology in order to weave between the axons that cross the radial path.

A third possibility is that proneural genes, expressed at the last RG division, induce the expression of proteins that cause pin-like cells to detach from the VZ and become multipolar (Itoh et al., 2013b). For example, induction of **Scratch** down-regulates **E Cadherin** (ECad, Cdh1), allowing separation of apical junctional complexes (Itoh et al., 2013a). **p27kip1**, **Dcx** and **Rnd2** are other examples. Dcx and Rnd2 seem to be regulated by cell-intrinsic factors and are considered further in Box 1. p27 is considered further here because its expression may be dynamically regulated by signaling through gap junctions (Liu et al., 2012).

**p27** is well known for regulating the cell cycle, but it also inhibits the small GTPase **RhoA** and regulates microtubules, thereby increasing cell motility (Besson et al., 2004, 2008; Godin et al., 2012). p27 knockout inhibits neuron migration from the VZ to the IZ, which is rescued by a mutant that lacks the N-terminal, cell cycle kinase regulatory function but retains its C-terminal, RhoA-binding region (Nguyen et al., 2006). Surprisingly, p27 expression in MP cells requires **Connexin 43** (Cx43): p27 expression is inhibited by Cx43 knockdown and increased by Cx43 over-expression (Liu et al., 2012). Knockdown of Cx43 inhibits IZ entry, with a consequent decrease in MP cells (Elias et al., 2007; Liu et al., 2012). Cx43 is best known as a component of gap junctions, aqueous channels for cell-cell communication. In the CP, however, Cx43 provides adhesion between BP neurons and RG, thereby enabling locomotion (Elias et al., 2007). Empirically, one would not expect that MP entry would require cell-cell adhesion. Indeed, MP entry requires Cx43’s channel function and C terminus, suggesting that gap junctions are involved (Liu et al., 2012). Cx43-dependent cell-cell communication could prime the p27 gene for induction during cell migration. The communication could occur between cells in the SVZ or between RG progenitors. Indeed, Cx43 is also needed for gap junction communication between progenitors and for interkinetic nuclear movement in the VZ (Liu et al., 2010). Therefore, gap junction communication between RG progenitors could be permissive for p27 gene induction by proneural genes when neuroblasts reach the IZ.

**p27** is also required later, for MP migration and exit from the IZ. A detailed study from Kawauchi et al. (2006) showed that acute knockdown of p27 with shRNA arrests neurons in the IZ. Cells in the lower IZ arrest with rounded morphology and reduced processes but those in the upper IZ have a clear radial process, suggesting defects in MP migration and locomotion of BP cells out of the IZ. The authors found that p27 inhibits phosphorylation of **cofilin**, an actin-severing protein that is inhibited by phosphorylation (i.e., p27 stimulates actin severing) (Kawauchi et al., 2006). This function of p27 is critically dependent on phosphorylation of p27 by **Cdk5** at Ser10, which protects p27 from proteasomal degradation and increases p27 levels. p27 likely inhibits cofilin phosphorylation by inhibiting **RhoA**, a known activator of the cofilin kinase, **LIMK** (Kawauchi et al., 2006). Therefore, Cdk5 and p27 together promote IZ exit by activating cofilin. Curiously, however, excess active cofilin also inhibits IZ exit (Kawauchi et al., 2006), suggesting that the balance between active and inactive cofilin is critical.

## Axon induction

MP cells extend axons during the random migration phase, before they reorient towards the pia and begin radial migration (Hatanaka et al., 2004; Barnes and Polleux, 2009; Hatanaka and Yamauchi, 2013; Namba et al., 2014; Figure 2, stage 2B). Axon extension is first detected in the lower IZ, which contains corticofugal efferents (Hatanaka et al., 2009; Namba et al., 2014). This suggests that signals from corticofugal axons may induce or stabilize axons. Corticofugal axons express the homophilic adhesion molecule **TAG1** (Cntn2), which is absent from thalamocortical axons in the upper IZ (Fukuda et al., 1997). Nascent axons produced by MP cells also express TAG1 and align with the corticofugal axons (Namba et al., 2014). TAG1 knockdown in the MP neurons inhibits axon formation and inhibited radial migration. The functional domains of TAG1 that are required for homophilic binding in trans *in vitro* are also required cell-autonomously for axon outgrowth. These results suggest that signals from TAG1 in the environment induce and stabilize the nascent axon by homophilic interactions. TAG1 is linked to the cell surface by a C-terminal glycosyl-phosphatidyl-inositol (GPI) moiety and is localized to lipid rafts, where it potentially activates Src-family kinases. Indeed, Namba et al show that the Src family kinase (SFK) **Lyn** and small GTPase **Rac1** are likely involved in stabilizing axons downstream of TAG1 (Namba et al., 2014).

In addition to TAG1, several receptor tyrosine kinases (RTKs) regulate axon induction *in vivo*. The neurotrophin receptors **TrkB** (Ntrk2) and **TrkC** (Ntrk3) and ligands **BDNF** and **NT3** are expressed in the VZ/SVZ. Trk inhibitors inhibit axonogenesis and migration out of the IZ in slice cultures (Ip et al., 2012). Also, sequestering neurotrophin ligands by expressing dominant interfering mutants of both TrkB and TrkC by *in utero* electroporation inhibited axon outgrowth and increased the percentage of MP neurons in the IZ (Nakamuta et al., 2011). TrkB knockout causes cortical lamination defects but axon defects were not noted, perhaps due to compensation by TrkC (Medina et al., 2004). Injection of BDNF or anti-BDNF antibodies into the ventricle stimulates or inhibits neuron migration, respectively (Fukumitsu et al., 2006). However, injected BDNF accelerates the entire neurogenic and migration process, so neurons born on a given day arrive more quickly at the top of the CP, settle in a lower layer position, and express earlier fate markers than usual. Taken together, these experiments suggest that neurotrophins probably stimulate axonogenesis and radial migration independently of neurogenesis, but cause and effect are not completely clear. Another RTK, **Kit**, is also expressed in migrating neurons and knockdown inhibits axon outgrowth and delays radial migration, although the mechanism remains to be determined (Guijarro et al., 2013).

Axon induction by BDNF *in vitro* requires **phospholipase Cγ**, **calcium**, and calcium-calmodulin regulated kinase kinase (**CaMKKα; Nakamuta et al., 2011**). Dual inhibition of TrkB and TrkC or knockdown of phospholipase Cγ or CaMKKα inhibited axon outgrowth and radial migration *in vivo*, suggesting that signaling through phospholipase Cγ and CaMKKα is important. An alternative mechanism involves α2-chimerin (Ip et al., 2012). α2-chimerin is a Rho GAP (GTPase activating protein) that contains an SH2 domain through which it binds to BDNF-stimulated TrkB (Ip et al., 2012). α2-chimerin knockdown at E14 stalls neurons in the MP stage. Rescue experiments showed that migration requires the SH2 domain but not GAP activity. α2-chimerin-inhibited neurons have abnormally high levels of dephosphorylated (active) **CRMP2**, a protein that stabilizes microtubules. This suggests that α2-chimerin-stimulation of CRMP2 phosphorylation may regulate migration. Consistent with this, migration of α2-chimerin-deficient cells was rescued by over-expressing wildtype or phospho-mimetic CRMP2 but not a non-phosphorylated mutant. Other studies have shown that CRMP2 is needed for the MP-BP transition, consistent with the idea that CRMP2 phosphorylation is needed to remodel the microtubule cytoskeleton (Sun et al., 2010). However, axon formation requires **Cdk5** and **axin** to inhibit **GSK3β** and thereby reduce phosphorylation of CRMP2 (Fang et al., 2011). This implies that CRMP2 is dephosphorylated during axon formation and re-phosphorylated when a radial leading process is stabilized. Nevertheless, there is no evidence that CRMP2 phosphorylation state changes during axon formation or migration. Indeed, the immunoreactivity of phospho-CRMP2 relative to total CRMP2 is constant through the IZ and CP (Ip et al., 2012). More study of specific phosphorylation sites and subcellular localization of CRMP2 may help resolve the mechanism.

## The coordination of axon induction with the transition from random to radial migration

The appearance of an axon before random migration ends suggests the possibility that axon formation is a prerequisite for starting radial migration (Figure 2, stage 2C). The TAG1 results are interesting in this regard. As mentioned above, TAG1 knockdown inhibits both axonogenesis and radial migration (Namba et al., 2014). Since TAG1 is an axonal molecule, it is unlikely to regulate radial migration directly. Therefore, this study suggests that axon outgrowth may be required before radial migration starts. In addition, inhibiting TrkB, TrkC and Kit impaired radial migration as well as axon induction (Fukumitsu et al., 2006; Nakamuta et al., 2011; Ip et al., 2012; Guijarro et al., 2013), consistent with, but not proving, causality.

On the other hand, there is evidence that neurons can enter the CP without axons. Deleting the kinase **LKB1** (Stk11, Par4) in progenitors inhibited axon outgrowth but neurons still migrated out of the IZ and into the CP, albeit in reduced numbers and with abnormal, highly branched leading processes (Barnes et al., 2007). LKB1 mutation does not cause gross lamination defects (Morgan-Smith et al., 2014). However, different results were obtained when LKB1 was acutely knocked down with shRNA (Asada et al., 2007; Matsuki et al., 2013). Many LKB1-deficient neurons accumulated in the IZ with multiple axon-like processes, suggesting defects in polarization which impact the selection of a single axon and migration (Asada et al., 2007). Acute knockdown of two LKB1 co-activators, **Stk25** and **STRADα**, also inhibited axon outgrowth and delayed cells in the IZ (Matsuki et al., 2010, 2013; Orlova et al., 2010). However, germline knockout of Stk25 did not affect layering or axonogenesis (Matsuki et al., 2013). The results are consistent with important roles for Stk25-STRADα-LKB1 in axon outgrowth and radial migration, but there may be bypass mechanisms that come into play when these proteins are absent. Overall the question of whether axonogenesis is required before a multipolar cell can stabilize a pia-directed leading process and start radial migration remains unanswered.

## Unc5D regulates the transition to radial migration

**Unc5D** is a co-receptor for **Netrins**, forming a complex with **DCC**/Unc40 and modulating DCC signaling. Unc5D expression is highest in the IZ (Sasaki et al., 2008). Transcription factor **FoxG1** represses Unc5D expression (Miyoshi and Fishell, 2012). FoxG1 is expressed in the VZ/SVZ and CP but is reduced in the IZ. Thus Unc5D increases when cells start MP migration and decreases when cells enter the CP. This suggests that the decline in Unc5D may trigger radial migration. However, inhibiting Unc5D expression (by over-expressing FoxG1) delayed the MP-BP transition, and co-over-expression of FoxG1 and Unc5D rescued normal migration. This suggests that the MP-BP transition requires high levels of Unc5D but the disappearance of Unc5D does not define the timing. In other systems, Netrin can bind to DCC in the absence of Unc5D, but the responses to DCC and Unc5D-DCC are different. DCC is expressed continuously during neuron migration. This means that MP cells have Unc5D-DCC complexes and Unc5D-deficient cells (which arrest in the IZ) have only DCC. This suggests that free DCC might inhibit IZ exit. Indeed, over-expressing DCC delays MP exit, which is rescued by co-expressing Unc5D (Miyoshi and Fishell, 2012). This suggests that MP exit may require DCC repression by Unc5D as a permissive event.

## Reelin and N-cadherin as permissive signals for radial polarization

**Reelin** is a secreted protein that regulates neuron migration in many brain regions (Honda et al., 2011). Full-length Reelin is most abundant where it is made in the MZ, but it is also cleaved and active fragments diffuse through the CP to the IZ (Jossin et al., 2007; Uchida et al., 2009). Genetic deletion of Reelin interferes with migration at several steps (Honda et al., 2011). Over-expressing dominant-negative Reelin receptors induces delay in the MP phase, with little or no inhibition of axon outgrowth (Jossin and Cooper, 2011).

Signaling proteins that are activated by Reelin are also required for radial polarization of MP cells. *In vitro*, Reelin stimulates a pathway including tyrosine phosphorylation of **Dab1** by **Src** and **Fyn**, recruitment of **Crk/CrkL** adaptors and **C3G**, a guanine nucleotide exchange factor (GEF) for **Rap1**, and increased GTP binding to Rap1 (Cooper et al., 2008). Some of these signaling proteins are also important in the MP-BP transition. For example, Dab1 knockdown inhibits migration in the IZ (Young-Pearse et al., 2007). Src and Fyn, the Crk/CrkL binding sites on Dab1, and the Crk and CrkL genes, are all required for normal lamination, but MP migration was not analyzed (Park and Curran, 2008; Feng and Cooper, 2009). C3G mutants arrest in the IZ with MP morphology and decreased Rap1GTP, implicating C3G and Rap1 (Voss et al., 2008). *In utero* electroporation with dominant-interfering Reelin receptors or Rap1A inhibitors also delays radial polarization, and over-expressed Rap1A partly rescues Reelin-inhibited MP cells, suggesting that Rap1A activation is necessary and partly sufficient for polarization (Jossin and Cooper, 2011). Rap1GTP regulates GEFs for the Ras-family member **RalA**, which regulates exocytosis, as well as Rho-family members **Rac1** and **Cdc42**, which regulate the actin cytoskeleton. Inhibition of Rac1or Cdc42 causes arrest of MP cells in the IZ (Kawauchi et al., 2003; Konno et al., 2005; Jossin and Cooper, 2011). Inhibition and rescue experiments implicated RalA, RalB, Rac1 and Cdc42 in Rap1-dependent radial polarization, suggesting that exocytosis and the actin cytoskeleton are involved (Jossin and Cooper, 2011).

The importance of exocytosis for radial migration is reinforced by the finding that a primary role of Reelin in the IZ is to induce surface traffic of **N-Cadherin** (NCad, Cdh2) (Jossin and Cooper, 2011). Rap1- or RalA-inhibited neurons have decreased cell-surface NCad and their migration is rescued by NCad over-expression. Dominant-interfering NCad inhibits MP exit (Kawauchi et al., 2010; Jossin and Cooper, 2011). The results suggest that Reelin regulates Rap1, RalA and other small GTPases to upregulate NCad, and that surface NCad is required to sense direction signals for radial migration and the MP-BP transition. NCad is not needed for locomotion of BP neurons, but is required again later, when Reelin regulates the final positioning of neurons at the top of the CP (Franco et al., 2011; Gil-Sanz et al., 2013).

It is unclear whether Reelin induces radial migration or is permissive. *In vivo*, NCad levels are high throughout the IZ, dependent on Reelin (Jossin and Cooper, 2011), so Reelin may induce NCad translocation to the cell surface throughout the period of MP migration. Moreover, Reelin is unlikely to be a direction signal: Reelin produced in the VZ or soaked into a brain slice can substitute for Reelin from the MZ (Magdaleno et al., 2002; Jossin et al., 2004). Therefore, Reelin may prime cells to sense another molecule that provides a direction signal and triggers radial migration as an instructive cue (Jossin and Cooper, 2011). Reelin is also required for correct radial orientation of other types of neurons (Landrieu and Goffinet, 1981; Nichols and Olson, 2010; Schneider et al., 2011; O’Dell et al., 2012), but in these situations it is also not clear whether it is instructive or permissive.

## Sema3A may induce and orient the leading process

**Plexin** (Plx) and **Neuropilin** (Nrp) family proteins are co-receptors for secreted **Semaphorins**, such as Sema3A. Importantly, Sema3A is secreted at the top of the CP and forms a gradient through the cortex. It is thus ideally suited to be a chemotactic factor for radial migration.

Seminal studies from Polleux and Ghosh showed that Sema3A orients cortical neurons so their dendrites grow towards the pia and axons towards the ventricle (Polleux et al., 1998; Polleux and Ghosh, 2002). Sema3A acts directly as an instructive signal by inhibiting axons and promoting dendrites (Shelly et al., 2011). Since the leading process of radially migrating neurons later develops into the dendritic tree, Sema3A may similarly induce the radial leading process on MP neurons. Indeed, Azzarelli et al found that MP neurons accumulate in the IZ when PlxB2 is inhibited (Azzarelli et al., 2014). Similarly, knockdown or Cre-mediated deletion of Nrp1, PlxA2, PlxA4 or PlxD1 increased the number of neurons trapped in the white matter and lower layers after birth, consistent with inhibition of MP migration (Chen et al., 2008). *In vitro*, Sema3A attracts neurons when added to one side of a slice culture or to one side of a porous membrane (Chen et al., 2008). This suggests that the gradient of Sema3A could provide a direction signal for radial migration, perhaps by stabilizing the leading process. However, it should be noted that cortical lamination is normal in Nrp1 mutant mice, at least up to E14.5 when embryonic lethality precludes analysis (Hatanaka et al., 2009). *In utero* expression of Nrp1/2 inhibitors at E12.5 or E15.5 also had no major effects on migration, although axon pathfinding was impaired (Hatanaka et al., 2009). It is not clear how to reconcile these findings.

The downstream signaling from Plx and Nrp receptors is currently unclear. However, PlxB2 may induce radial migration by stimulating** RhoA**. RhoA has a general role in stabilizing filamentous actin and inducing actomyosin contraction. PlxB2 binds to a RhoA activator, **PDZ-RhoGEF**/LARG. Removing PlxB2 lowered RhoA activity and over-expressing RhoA partly rescued the migration of PlxB2-deficient neurons. The C terminus of PlxB2 is required to bind PDZ-RhoGEF and rescue migration of PlxB2-deficient cells. This suggests that PlxB2 activates RhoA through PDZ-RhoGEF and that RhoA activity is needed for radial migration. However, there is currently no evidence that RhoA activity increases when radial migration starts. Cyclic nucleotides may also be involved (Liu et al., 2004). One attractive possibility is that Sema3A, coming from the top of the cortex, contacts the upper side of a multipolar cell, activates PlxB2, Nrp1 and RhoA locally, inducing and stabilizing a pia-directed process. However, testing this model will require improved RhoA activity reporters and high-resolution live-imaging of MP neurons as they reorient radially in the IZ.

## Other signaling molecules required for radial migration: instructive or permissive?

The last decade has seen the discovery of a plethora of proteins that are required for or inhibit the MP-BP transition. In most cases, authors have used loss of function (knockdown or knockout) and gain of function (in most cases, over-expression) experiments to demonstrate necessity and sufficiency of specific proteins for radial migration. Some of the proteins, including LIS1 (Pafah1b1), Ndel1, dynamin, FilaminA and Doublecortin (DCX), were reviewed thoroughly previously, and will not be considered here (LoTurco and Bai, 2006; Ayala et al., 2007). Progress in understanding other genes that regulate radial migration is summarized in Box 1. However, in nearly every case, it remains unclear whether they are permissive or whether their activation (or inhibition) sets the start time for radial migration and transformation to BP morphology.

## Lateral migration of MP cells and the radial unit hypothesis

Once MP migration starts, neurons migrate laterally as well as up and down. The horizontal movement needs to be reconciled with the radial unit hypothesis (Rakic, 2000). This postulates that cells move radially from the VZ to CP, allowing the protomap of functional areas in the VZ to be relayed into the CP. Radial migration means that sister neurons are arranged in “mini-column” units (reviewed by Gao et al., 2013). Consistent with strictly radial migration, locomoting neurons are generally detected within 1 or 2 cell diameters (10–20 μm) of their sister RG (Noctor et al., 2001, 2002). After migration is complete, neurons preferentially form electrical and chemical synapses with their sisters in other layers (Yu et al., 2009, 2012). If neurons wander laterally during MP migration, how do they remain close to their parental RG and how do they find their sisters when they are forming circuits?

One way that the MP cells could remain close to their parent RG would be to follow a random walk. Given a MP velocity of 2–4 μm/h (Tabata and Nakajima, 2003; Jossin and Cooper, 2011; Dimidschstein et al., 2013) and travel time of 24 h, a neuron could travel 50–100 μm in a straight line but just a few cell diameters (10–20 μm) if it changes direction every hour. However, the distance would be expected to increase with the time spent migrating. The relationship between horizontal spread and time spent migrating has not been established, but Sanada et al. (2004) found that the lateral dispersion of neurons was reduced from 16 μm to 4–8 μm when Reelin signaling was inhibited, even though the cells had presumably spent more time in the IZ and had progressed less far into the CP. This is the opposite of what would be expected if lateral dispersion is passive.

**Figure 3:**
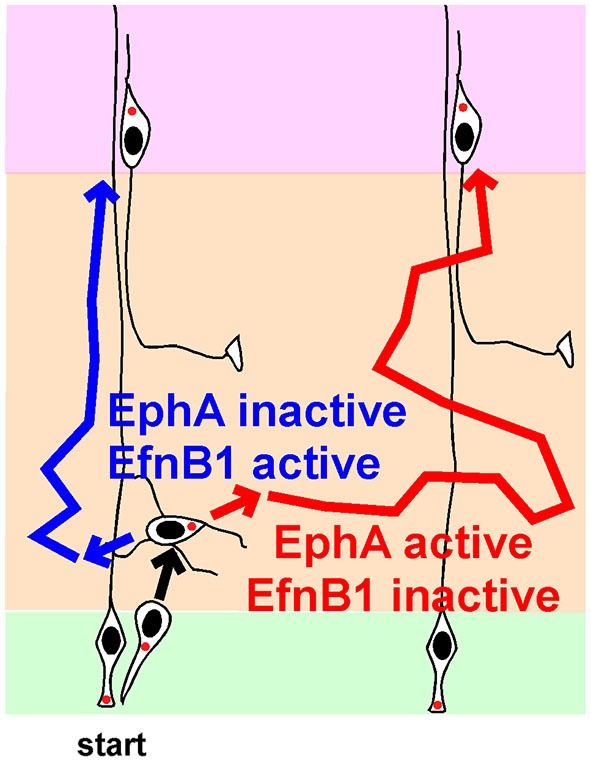
**Regulation of lateral movement by Ephs and Ephrins (Efns) during multipolar migration.** Newborn neurons starting from one radial glia progenitor (RG) can follow the blue path, and position radially above their starting point, or follow the red path, and disperse laterally, depending on the strengths of signaling from EphA-EfnA and EphB-EfnB signals. See text for details and references.

An alternative hypothesis is that the direction of MP migration is not random, but is controlled by signaling molecules. Indeed, there is now excellent evidence that horizontal movement of MP cells is regulated by Ephrin (Efn) and Eph family proteins (Figure 3).

Efns are cell surface proteins that activate Eph family RTKs on other cells and generate “forward signals”. Reciprocally, an Eph can activate “reverse signaling” into an Efn-expressing cell. EfnA molecules are glycosylphosphatidylinositol-linked cell surface proteins that bind EphA family members. EfnB molecules are transmembrane proteins that bind EphB family members. Efns and Ephs regulate cell sorting in many developmental systems (Fagotto, 2014).

Several **EfnA** and **EphA** genes are expressed in the developing cortex (Torii et al., 2009). In the IZ, EfnAs are expressed more medially and less laterally. Their ligands, EphA RTKs, are expressed in the IZ in a counter gradient, higher laterally than medially. This expression pattern suggests that EfnA or EphA activity could regulate lateral dispersion, e.g., if cells of the same EfnA:EphA ratio sort together. This hypothesis was tested by studying a EfnA2/3/5 triple mutant (Torii et al., 2009). At birth, there were no gross effects on cortical development or layer order, but the cortical layers contained undulations. When clones were labeled at E12.5 and examined at E14.5, neurons in wildtype clones averaged 20 μm from the RG while they were only 10 μm distant in the triple knockout. Live imaging confirmed that tangential migration is inhibited when EfnAs are absent.

Somewhat paradoxically, over-expression of an EfnA ligand, EphA7 or EphA4, caused over-expressing neurons to segregate from non-expressing neurons in the IZ (Torii et al., 2009). Each cluster contained neurons from several clones. Later, these clusters gave rise to cortical columns of over-expressing cells separated from other columns by normal cells. Column formation required EphA cytoplasmic domains, suggesting forward signaling from EfnA to EphA. Live imaging showed that this increased clumping is actually due to greater dispersion of EphA over-expressing neurons while they are migrating in the MP zone. Thus, the live imaging is consistent with the EfnA triple knockout, in that decreased EfnA (and decreased EphA forward signaling) decreases lateral movement in the IZ and increased EphA expression (and presumably EphA forward signaling) increases lateral movement of MP cells in the IZ. The results are consistent with MP cells choosing their lateral position based on the strength of EphA forward signaling (Figure 3, compare blue and red routes in absence and presence of EphA signaling).

In contrast with EfnAs stimulating lateral movement by forward signaling, endogenous EfnBs may inhibit lateral movement by reverse signaling. Over-expression of **EfnB1** at E13 in post-mitotic neurons caused over-expressing neurons to segregate away from non-expressing neurons (Dimidschstein et al., 2013). Despite the marked change in lateral distribution, layering was normal. There was no evidence for increased homotypic adhesion. Rather, the MP cells had shorter processes, perhaps indicating reduced migration in the IZ. This effect required the extracellular and cytoplasmic domains of EfnB1, consistent with reverse signaling from EphBs. The C terminus of EfnB1 binds a Rho-family GEF, **P-Rex1**. The abnormal clustering induced by over-expressing EfnB1 required the EfnB1 C terminus, P-Rex1 and **Rac3**, a little studied Rho-family member that is a primary substrate for P-Rex1 and is implicated in opposing Rac1 and reducing neurite number (Dimidschstein et al., 2013). This suggests that high levels of EfnB1 activity reduce MP migration in the IZ, reducing lateral spread (Figure 3). Consistently, deletion of EfnB1 increased the number of MP cell neurites and the speed of movement in the IZ. However, lateral spread of deleted clones was only increased by ~20%. It is possible that combined disruption of EfnB1 with EfnB2 and EfnB3 would give a more severe dispersion and confirm the importance of endogenous EfnB family members in regulating horizontal movement.

## The varied duration of MP migration may affect lamination

**Figure 4:**
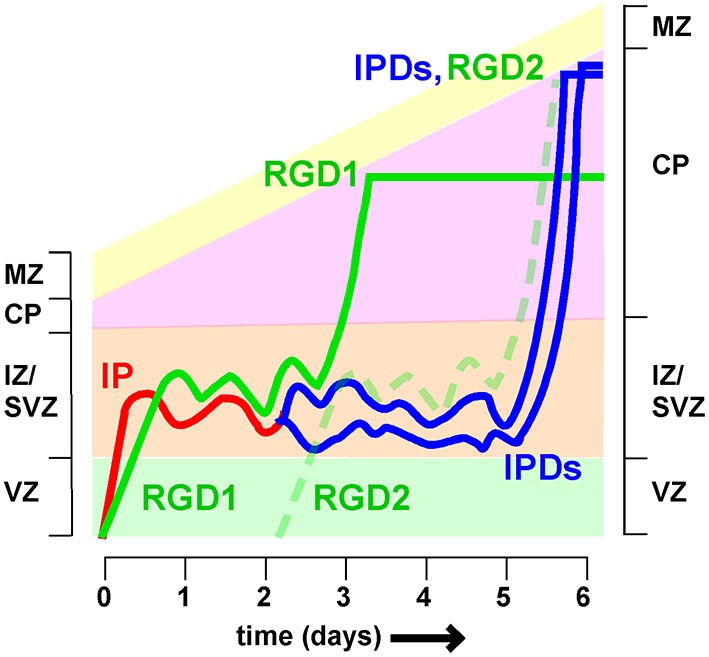
**Different timing of intermediate zone exit influences cortical lamination** Neurons spend variable times migrating in the intermediate zone (IZ). Postmitotic neurons derived directly from radial glia (i.e., radial glia daughters, RGD) spend approximately 2 days in the IZ before entering the cortical plate (CP). Intermediate progenitors (IP) divide to create intermediate progenitor daughters (IPDs), for a total time of approximately 5 days in the IZ. As a result RGD born on day 0 (RGD1) arrive at the top of the CP 2–3 days before IPDs whose mother IP also left the VZ on day 0. As a result the IPDs layer above RGD1, and co-layer with RGD2, born 2 days later. If neuron fate is fixed at the last RG division, then this would cause mixing of neurons of different fates in the same layer.

Time-lapse recordings and birthdating studies indicate that MP migration lasts for at least a day in the mouse, but the timing is very heterogeneous (Noctor et al., 2004; Westerlund et al., 2011). Neurons that spend longer in the MP phase are expected to arrive later at the top of the CP than neurons that travel more rapidly. This means they will enter higher cortical layers. Since neuron fate is thought to be fixed at the last division of the RG progenitor, different transit times are expected to blur the discrete layering of neuronal subtypes that characterizes the mature cortex (Molyneaux et al., 2007; Figure 4). It remains unclear whether or how the varied timing of MP migration is compensated at other stages of development.

One major source of variability is that post-mitotic neurons and IP enter and leave the IZ at different rates (Tabata et al., 2009). Radial glia daughters (RGDs) that are post-mitotic move slowly (>10 h) from the VZ to the SVZ/IZ but have a relatively short MP phase, entering the CP between 36 and 60 h (Figure 4, see RGD1). In contrast, IPs move quickly (<10 h) to the SVZ/IZ but then divide. The intermediate progenitor daughters (IPDs, Figure 3) become multipolar and continue migrating. They enter the CP >60 h after the RG division (Tabata et al., 2009). IP daughters are thus expected to layer above their “aunts” (RG daughters) at the top of the CP (Figure 3). Does this mean that IP daughters switch to a later fate than RG daughters or does this lead to broadening and mixing of cortical layers?

One idea is that fates are not fixed at the last division of the RG progenitor but remain plastic (Fishell and Hanashima, 2008). Cell fate may be determined at the last division, whether it occurs in the VZ or SVZ/IZ. In this way, the fates of IP daughters and RG daughters entering the same lamina could be equivalent. Indeed, manipulation of FoxG1 not only delays cell exit from the IZ, so neurons shift to a higher lamina, but also adjusts the neuron fate to match the new position (Miyoshi and Fishell, 2012; Toma et al., 2014). Further testing of this hypothesis during normal development requires approaches for distinguishing IP daughters from RG daughters, and testing whether they co-layer and express the same markers (Tabata et al., 2009).

## Conclusions

Amazing technical refinements in mouse genetics, imaging, *in utero* manipulation, and slice culture methods have led to the discovery of a large number of genes that regulate MP migration, axonogenesis, and direction of movement. Many of the genes have been subjected to epistasis analysis, implying causal relationships and sequences of contingent events. Some protein activities are regulated by external signals, while others may be “hard-wired” by cell intrinsic mechanisms or transcription changes. Despite dramatic progress, it is still unclear how these different processes fit together. We have limited understanding of how different pathways interact and exactly when and where in the cell signaling occurs. Analysis is extremely challenging given the asynchrony of the cell population, the small size of the cells, and the difficulties of imaging signaling events within living tissue. However, given the continuous development of new technology, we can be optimistic that these obstacles will be overcome.

## Conflict of interest statement

The author declares that the research was conducted in the absence of any commercial or financial relationships that could be construed as a potential conflict of interest.
